# An Improved Deep Residual Network Prediction Model for the Early Diagnosis of Alzheimer’s Disease

**DOI:** 10.3390/s21124182

**Published:** 2021-06-18

**Authors:** Haijing Sun, Anna Wang, Wenhui Wang, Chen Liu

**Affiliations:** 1 College of Information Science and Engineering, Northeastern University, Shenyang 110819, China; seamirror@126.com (H.S.); wenhuiwang@stumail.neu.edu.cn (W.W.); liuchenne@gmail.com (C.L.); 2 College of Information Engineering, Shenyang University, Shenyang 110044, China

**Keywords:** residual network, Mish, spatial transformer networks, non-local attention mechanism, Alzheimer’s disease

## Abstract

The early diagnosis of Alzheimer’s disease (AD) can allow patients to take preventive measures before irreversible brain damage occurs. It can be seen from cross-sectional imaging studies of AD that the features of the lesion areas in AD patients, as observed by magnetic resonance imaging (MRI), show significant variation, and these features are distributed throughout the image space. Since the convolutional layer of the general convolutional neural network (CNN) cannot satisfactorily extract long-distance correlation in the feature space, a deep residual network (ResNet) model, based on spatial transformer networks (STN) and the non-local attention mechanism, is proposed in this study for the early diagnosis of AD. In this ResNet model, a new Mish activation function is selected in the ResNet-50 backbone to replace the Relu function, STN is introduced between the input layer and the improved ResNet-50 backbone, and a non-local attention mechanism is introduced between the fourth and the fifth stages of the improved ResNet-50 backbone. This ResNet model can extract more information from the layers by deepening the network structure through deep ResNet. The introduced STN can transform the spatial information in MRI images of Alzheimer’s patients into another space and retain the key information. The introduced non-local attention mechanism can find the relationship between the lesion areas and normal areas in the feature space. This model can solve the problem of local information loss in traditional CNN and can extract the long-distance correlation in feature space. The proposed method was validated using the ADNI (Alzheimer’s disease neuroimaging initiative) experimental dataset, and compared with several models. The experimental results show that the classification accuracy of the algorithm proposed in this study can reach 97.1%, the macro precision can reach 95.5%, the macro recall can reach 95.3%, and the macro F1 value can reach 95.4%. The proposed model is more effective than other algorithms.

## 1. Introduction

Alzheimer’s disease (AD) is a common, irreversible, progressive neurological disease characterized by cognitive impairment, whereby the patient’s memory and thinking ability are slowly damaged over time [1,2]. AD is characterized by an insidious onset, slow progression, and irreversible course. Currently, there is no effective treatment that can reverse the damage caused by Alzheimer’s disease [3,4]. At present, most patients with clinically diagnosed AD are in the middle or advanced stage, which means that the optimal time for treatment has already passed. Mild cognitive impairment (MCI) is an intermediate state between normal function and AD. It refers to a mild impairment of cognitive and memory functions rather than dementia [5,6]. According to statistics, the conversion rate of people with MCI to AD is significantly higher than that of healthy people [7]. Accurate diagnosis early in the course of the disease may allow patients to initiate preventive and intervention measures to slow or stop the progression of the disease before irreversible brain damage takes place. Therefore, the early and accurate diagnosis of AD has important research significance [8,9].

At present, there are the following three categories of methods for the early diagnosis of AD: diagnostic methods based on clinical symptoms and cognitive function examination scales, biomarker-based detection methods, and neuroimaging-based detection methods [10,11,12]. The diagnostic method, based on clinical symptoms and a cognitive function examination scale, can be used in the quantitative assessment of cognitive impairment, and has the advantages of easy operation, low cost, and standardized diagnosis. However, due to the lack of objective evidence, it is easy for the diagnosis to be detrimentally influenced by non-pathological subjective factors, which inevitably lead to clinical misdiagnosis. [13,14,15]. A biomarker-based detection method is used to diagnose the disease and judge the disease stage by measuring biomarker levels in patients. For example, β-amyloid_1–42_ (Aβ_1–42_), total tau protein (T-tau), and hyperphosphorylated tau (P-tau), the core biomarkers of AD that are mostly used in clinical practice, are derived from CSF and have shown high sensitivity and specificity in the identification of AD patients. Through providing effective diagnosis of AD in its early or asymptomatic stages, this approach may buy time for patients, increasing the effectiveness of treatment and reducing the disease incidence. However, this method cannot be used as a routine detection method due to the difficulty and traumatic nature of obtaining samples for determination of these biochemical indicators [16,17]. This problem can be circumvented when using neuroimage-based diagnostic methods. High-quality medical images, such as those acquired using positron emission computed tomography (PET) and magnetic resonance imaging (MRI), can provide doctors with more sufficient and subtle disease information, help doctors make diagnoses more quickly and accurately, and greatly reduce the misdiagnosis efficiency [18,19]. PET scans are performed after a patient is injected with a radioactive substance to see if there are lesions. However, PET has the disadvantages of poor specificity, low resolution, inaccurate anatomical positioning, and high cost [20]. MRI can clearly show the structure and anatomy of the human brain, which is conducive to the measurement and study of changes during brain atrophy in AD [21,22]. At present, the MRI diagnosis of AD is mainly based on subjective image reading by imaging doctors. This method is time consuming, laborious, and subjective, which will affect the accuracy in making judgments regarding the disease course. Hence, how to address these issues by using a computer to automatically and accurately classify MRI, and then identify MCI and AD has become a hot research topic in recent years [23,24].

In the past ten years, deep learning and machine learning methods have achieved great success in the fields of speech recognition, computer vision, and image and video analysis [25]. More and more studies have applied convolutional neural network (CNN) combined with MRI imaging for the early diagnosis of AD [26]. The traditional CNN network is usually composed of an input layer, hidden layer, and output layer in series. The input layer is responsible for receiving input data [27]. The hidden layer is generally composed of multiple convolutional layers and pooling layers. Its function is to extract the layered features of the input images. This hierarchical structure can gradually extract the high-level features in the image. However, these methods have the following disadvantages: given the depth of the layers in the traditional CNN network structure, excessively deep networks can actually reduce the accuracy of classification to a certain extent [27,28]. Moreover, traditional CNN lacks invariance to the affine transformation of the image. This defect is caused by the CNN default sampling method (matrix sampling). The convolutional layer of traditional CNN cannot satisfactorily extract the long-distance correlation in the feature space. In order to solve the above problems, an improved deep residual network (ResNet) model, combining spatial transformer networks (STN) and a non-local attention mechanism, is proposed for the early diagnosis of AD [29,30,31].

In this study, our key contributions are given below:

1. We propose a new ResNet model where a new Mish activation function is selected in the ResNet-50 backbone to replace the Relu function;
2. The STN is introduced between the input layer and the improved ResNet-50 backbone. This enhances the spatial invariance of the model;
3. A non-local attention mechanism is introduced between the fourth and fifth stages of the improved ResNet-50 backbone.

## 2. Related Work

In this section, we review studies related to the early diagnosis of AD. With the increasing attention given to MCI, more and more researchers have proposed new MCI prediction methods [32].

There are diagnostic methods based on the clinical symptom and cognitive function scale. Several AD screening scales commonly used in clinical practice include the clock-drawing test (CDT), mini-mental state examination (MMSE), Montreal cognitive assessment (MOCA), and Alzheimer’s disease assessment scale (ADAS-COG), among others. [33,34,35]. Brodaty H. et al. [36] argued that a clock map is a very effective test screening measure for detecting mild or moderate AD in the clinical population, with very low false-negative and false-positive rates. Pozueta A. et al. [37] proposed that a combination of MMSE and CVLT-LDTR could distinguish PR-AD and S-MCI at the baseline. The analysis of these two neuropsychological predictors is relatively short and may be easily accomplished in a non-specialist clinical setting. Zainal N. et al. [38] proposed that ADAS-COG, which is widely used in clinical trials, may be suitable for an Asian cohort, and is useful for detecting MCI and mild AD. Roman F. et al. [39] proposed that Argentina-type MBT and MMSE were significantly correlated with memory cells and that they were effective tools for detecting MCI. The working characteristics of the MBT are very suitable, more so than those of other commonly used tests for detecting MCI. Carlew A. et al. [40] proposed that detection by MMSE is significantly affected by the disease course, while in the case of MOCA, severe MCI results in insignificant changes. Although statistically significant, the actual clinical significance of the changes in MOCA is unclear. The growing use of MOCA requires further research to understand what constitutes clinically significant changes and whether it is appropriate to track cognitive trajectories.

There are diagnostic methods based on biomarker detection; cerebrospinal fluid (CSF) biomarkers Aβ_1-42_, T-tau, and P-tau are well-validated, and are being increasingly used in clinical practice as tools for the affirmative diagnosis of AD [41]. The long-term stability of core CSF biomarkers in patients with AD provides further support for their use in clinical studies and treatment monitoring in clinical trials [42]. Michael E. et al. [43] proposed that CSF Aβ_1-42_ showed the best diagnostic accuracy among the CSF biomarkers. At a sensitivity of 85%, the specificity in differentiating AD dementia from other diagnoses ranged from 42% to 77%. Geijselaers S. et al. [44] provided further evidence of the relationship between brain insulin signaling and AD pathology. This also highlights the need to consider sex and the APOE ε4 genotype during assessment. Gs A. et al. [45] proposed that blood, urine, saliva, and tears have yielded promising results, and several new molecules have been identified as potential brain biomarkers thanks to the development of new ultra-sensitive techniques. In this review, the authors discuss the advantages and limitations of classic CSF biomarkers for AD, as well as the latest prospects for new CSF candidate biomarkers and alternative substrates. Fossati S. et al. [46] found that plasma tau is higher in AD independently from CSF-tau. Importantly, adding plasma tau to CSF tau or P-tau improves the diagnostic accuracy, suggesting that plasma tau may represent a useful biomarker for AD, especially when added to CSF tau measures. Abe K. et al. [47] found that the present serum biomarker set provides a new, rapid, non-invasive, highly quantitative, and low-cost clinical application for dementia screening, and also suggests an alternative pathway or mechanism by which AD causes neuroinflammation and neurovascular unit damage. Nabers A. et al. [48] used immune infrared sensors to measure the secondary structure distribution of amyloid beta (Aβ) and tau in plasma and cerebrospinal fluid as structure-based biomarkers of AD. In the first diagnostic screening step, structure-based Aβ blood biomarkers support AD recognition with a sensitivity of 90%. In the second diagnostic validation step, the combination of structure-based cerebrospinal fluid biomarkers Aβ and tau allowed the exclusion of false positives, with an overall specificity of 97%.

There are also neuroimage-based detection methods. Basheera S. et al. [49] used a CNN model with inception blocks to extract depth features from gray matter slices for the early prediction of AD. Ji H. et al. [50] mainly studied the early diagnosis of AD using convolutional neural networks. The gray matter and white matter image slices of MRI were used as classification inputs. After a convolution operation combined with the output of deep learning classifier, an ensemble learning method was adopted to improve classification. Tofail B. et al. [51] proposed constructing multiple deep two-dimensional convolutional neural networks (2D-CNNs) to learn various features from local brain images and combine these features with the final classification for AD diagnosis. Subramoniam M. et al. [52] proposed a method for the prediction of AD from MRI based on deep neural networks. The state of image classification networks, such as VGG, residual network (ResNet), etc., with transfer learning, show promising results. The performance of pretrained versions of these networks can be improved by transfer learning. A ResNet-based architecture with a large number of layers was found to give the best result in terms of predicting different stages of the disease. Hussain et al. [53] proposed a model based on 12-layer CNN to use brain MRI data for the dichotomization and detection of AD.

Among the abovementioned methods available for the early diagnosis of AD, the diagnosis method based on MRI has the advantages of non-invasiveness and non-radioactivity, and has become an indispensable technical tool in the clinical and scientific research of AD. In this study, ResNet-50 is used as the backbone network because of its simpler structure, and since the increase in identity mapping does not reduce network performance. The proposed method can extract more information from layers by deepening the network structure through deep ResNet [54,55]. 

## 3. Materials and Methods

### 3.1. Data Selection

The data used in this study come from ADNI (Alzheimer disease neuroimaging initiative) (http://adni.loni.usc.edu (accessed on 16 February 2020)) [56]. Generally, the dataset is divided into the following 3 categories: normal control (NC), mild cognitive impairment (MCI), and Alzheimer’s disease (AD). MCI is a major step in the transition from a normal to AD state. We screened a total of 515 samples, which were divided into 55 AD samples, 255 NC samples, and 205 MCI samples. The proportion of men and women in each category was roughly equal. MMSE mainly relies on experienced doctors to ask patients to obtain scale scores. The scale score is a continuous integer from 0 to 30. The higher the score, the healthier the patient, while the lower the score, the more severe the dementia. For NC, the MMSE score is 24–30 and the ADAS-Cog score is <12. For MCI, the MMSE score is 23–30 and the ADAS-Cog score is 7–17. For AD, its MMSE score is 20–26 and the ADAS-Cog score is 12–29 [57]. Information on the collected data is shown in Table 1.

### 3.2. Deep Residual Neural Network

**Figure 1:**
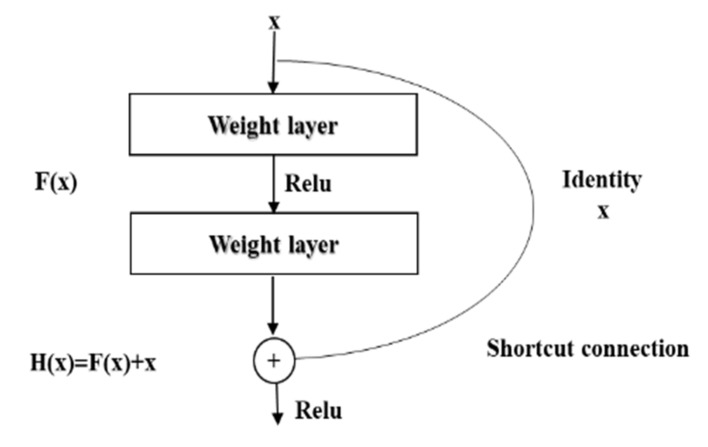
The residual block of the residual network.

ResNet was proposed by 4 Chinese scientists, including Kaiming He, from the former Microsoft Research Institute, and the proposal of deep ResNet is a milestone event in the history of CNN images. The residual module in the deep ResNet is shown in Figure 1 [58].

In the figure, x is weighted by the first layer, then F(x) + x is obtained after the non-linear variation in the Relu function and the weighting of the second layer. This is a linear stack, and the two layers constitute a residual learning module. The network composed of residual modules is called ResNet. The difference between the ResNet and the ordinary network is that the jump connection is introduced, which can help the information of the previous residual block flow into the next residual block without obstruction. The problem of vanishing gradient and degradation caused by too deep a network is avoided [58,59]. 

Since the Relu function often causes the permanent inactivation of neurons, these inactivated neurons will be occupied. Due to the computational resources involved, the ability to extract image features still needs to be improved. In order to make up for the deficiency of Relu, the new activation function Mish was selected to replace the function of Relu in the model. The Mish activation function is expressed as in Equation (1) [60], as follows: 

(1) $$f(x)=xtanh(ln(1+e^{x}))$$

The positive value of the Mish activation function can reach any height, avoiding saturation due to capping. Due to the smoothness of the Mish activation curve, better information can be penetrated into the neural network, resulting in better accuracy and generalization. As the depth of the network increases, Mish can better maintain accuracy. 

**Figure 2:**
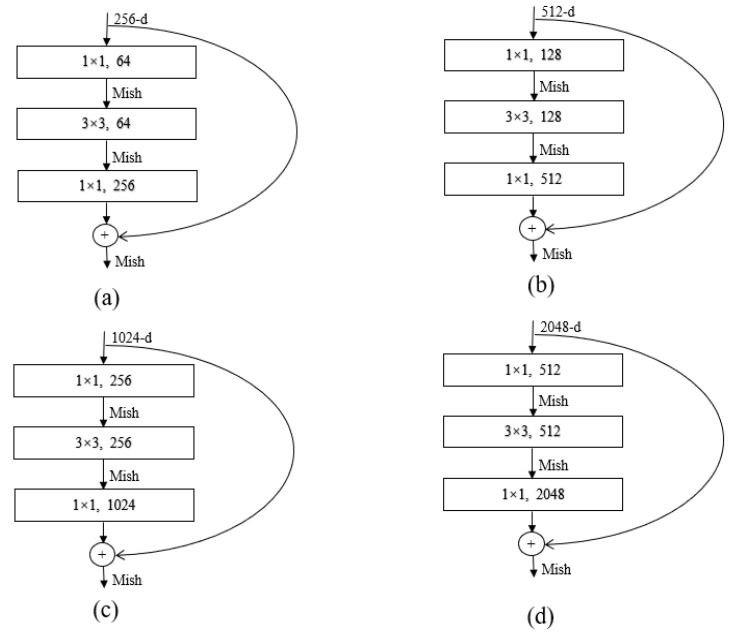
Bottleneck residuals module of different layers for the ResNet-50 architecture. (**a**) Stage 2, (**b**) stage 3, (**c**) stage 4, (**d**) stage 5.

In the deep ResNet-50, the bottleneck residual module is stacked with a 1 × 1 convolution, 3 × 3 convolution, and 1 × 1 convolution. The two l × 1 convolutions play the role of decreasing and increasing dimensions, respectively. The bottleneck residual module can greatly improve the computational efficiency and significantly increase the depth of the residual block. The introduction of more Mish activation functions can improve the representation ability of ResNet. The bottleneck residuals module of different layers for the ResNet-50 architecture is expressed in Figure 2 [58,59,60].

### 3.3. Spatial Transformer Networks (STN)

**Figure 3:**
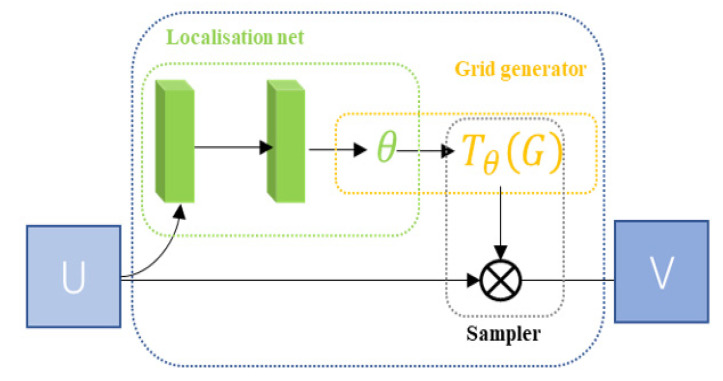
The STN module.

STN can adaptively perform spatial transformation. In the case of large spatial differences in the input data, this network can be added to the existing convolutional network to improve the accuracy of classification. The STN network consists of a localization network, a grid generator, and a sampler, as shown in Figure 3 [61].

Localization net: localization net is traditional CNN and this is the network used for the regression transformation parameter *θ*.

Grid generator: the grid generator generates a coordinate network corresponding to each pixel of the output image in the input image.

Sampler: a sampler uses the sampling network and the input element graph as an input, then inputs, then obtains the result after transforming the element graph.

After the input picture is passed through the STN module, the transformed picture is obtained, and the transformed picture is then input into the CNN network. Loss is calculated through the loss function, and the gradient is then calculated to update the *θ* parameter. Finally, the STN module will learn how to correct the picture [61,62].

### 3.4. Non-Local Attention Mechanism

**Figure 4:**
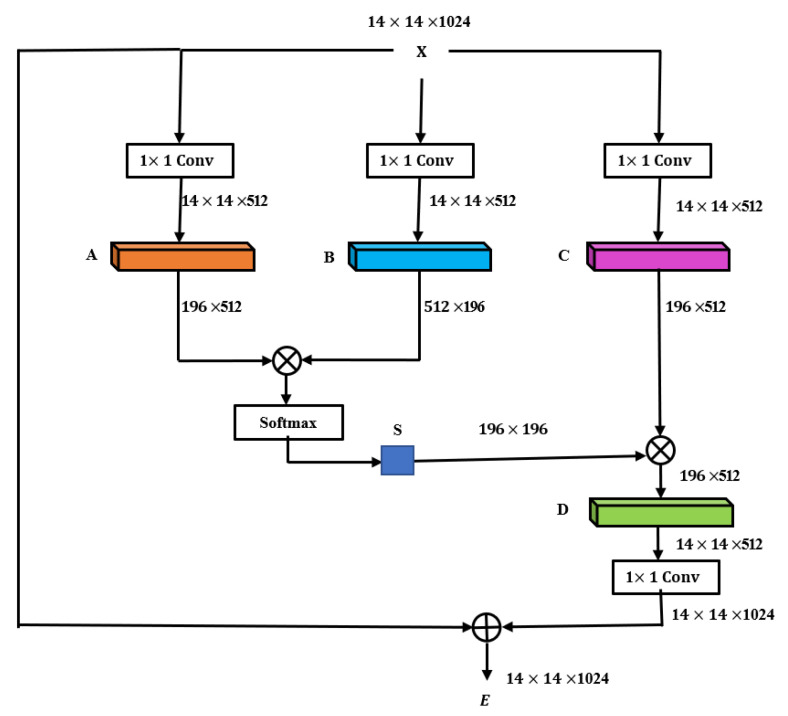
Non-local attention mechanism.

The attention mechanism is a general mechanism for information acquisition, which is applied to scenarios where a large number of sources are used to obtain specific critical information and avoid processing all the data. The non-local attention mechanism directly captures remote dependencies by calculating the interaction between any two locations, rather than being limited to adjacent points. The non-local attention mechanism is shown in Figure 4 [63].

Three feature images, A, B, and C, can be obtained through three 1 × 1 convolutional layers. A and B are multiplied to obtain *S* using softmax. Then, the product of *S* and *C* can be multiplied by the scale coefficient to obtain D, D can be reshaped to the original shape, and then *X* is added to obtain the final output *E*. We can see that the value of each position of *E* is a weighted sum of the original feature and each position. $E_{j}$ can be written as Equation (2) [63,64].

(2) $$E_{j}=\alpha \sum_{i=1}^{N}(S_{ji}C_{i})+X_{i}$$

### 3.5. Proposed Method

**Figure 5:**
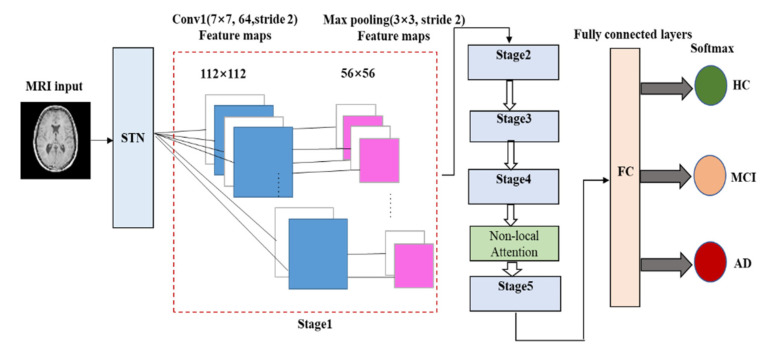
The framework of the proposed method.

In this paper, a new deep ResNet learning method that combines STN and the non-local attention mechanism is proposed. The model uses MRI slices of a large number of subjects to train the network, automatically learns image features, avoiding manual extraction, and then classifies the input images based on these features to obtain diagnosis results for the subject’s state. In this study, a new activation function Mish was selected to replace Relu in the traditional ResNet-50 model. This method could solve the problem of local information loss in ordinary CNN and can satisfactorily extract the long-distance correlation in feature space. The framework of the proposed method is shown in Figure 5 [58,59,60,61,62,63,64,65].

A local network is a network used in regression of transformation parameter *θ*. Its input is a feature image and its output is the spatial transformation of parameter *θ* through a series of hidden network layers. If a 2D affine transformation is required, *θ* is the output of a 6-dimensional (2 × 3) vector. The size of *θ* depends on the type of transformation applied.

A grid generator is used to build a sampling grid according to the predicted transformation parameters. It is the output of a group of points in the input image after sampling and transformation. What the grid generator actually obtains is a kind of mapping relation $T_{\theta }$ [62].

Assuming that the coordinate of each pixel of input image is ($x_{i}^{s}$, $y_{i}^{s}$), the coordinate of each pixel of output image is ($x_{i}^{t}$, $t_{i}^{t}$). The space transformation function $T_{\theta }$ is a two-dimensional affine transformation function. The corresponding relationship between ($x_{i}^{s}$, $y_{i}^{s}$) and ($x_{i}^{t}$, $y_{i}^{t}$) can be written as Equation (3), as follows:

(3) $$\left(\begin{array}{c} x_{i}^{s}\\ y_{i}^{s} \end{array}\right)=T_{\theta }\left(G_{i}\right)=A_{\theta }\left(\begin{array}{c} x_{i}^{t}\\ y_{i}^{t}\\ 1 \end{array}\right)=\left[\begin{array}{ccc} \theta_{11} & \theta_{12} & \theta_{13}\\ \theta_{21} & \theta_{22} & \theta_{23} \end{array}\right]\left(\begin{array}{c} x_{i}^{t}\\ y_{i}^{t}\\ 1 \end{array}\right)$$

In Equation (3), *S* represents the coordinate point of the input feature image, *T* represents the coordinate point of the output feature image, and $A_{\theta }$ is the output of the local network.

The sampler in STN uses the sampling grid and the input feature map as the input to produce the output. Additionally, it obtains the result after the feature map is transformed. Further, *n* and *m* will traverse all coordinates of the original graph *U*, and $U_{nm}$ refers to the pixel values of a point in the original graph *U*. Then, $x_{i}^{s},y_{i}^{s}$ denotes the coordinates of the corresponding point in the *U* graph to be found at the *i*th point in *V*. The denoted coordinates are those on the *U* graph. *K* denotes filling by different methods, usually using bilinear interpolation. The following Equation (4) is obtained [61,62]:

(4) $$V_{i}=\sum_{n}\sum_{m}U_{nm}max\left(0,1-\left|x_{i}^{s}-m\right|\right)max\left(0,1-\left|y_{i}^{s}-n\right|\right)$$

Integrating the STN module between the input and ResNet allows the network to automatically learn how to transform the feature map, thus helping to reduce the overall cost of network training. We locate the output value in the network, indicating how to transform each item of training data [61,62,63,64].

The non-local attention mechanism is embedded as a component in ResNet-50, and new weights are learned in transfer learning so that pretrained weights are not unavailable due to the introduction of new modules [63,65]. 

The architecture of the proposed method is shown in Table 2 [58].

**Figure 6:**
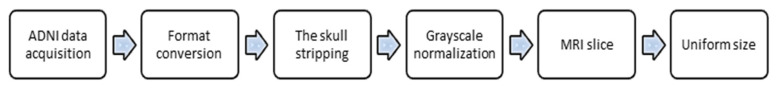
Preprocessing flow chart.

The environment of this experiment is a Linux system, which is designed and realized by the Keras framework, and the model is trained using the Adam optimization algorithm. The experiment steps are as follows:

(1) The experiment uses two-dimensional slices as training data, so it is necessary to slice the three-dimensional MRI coronal plane. In order to ensure that the input image size of the classifier is consistent, this experiment unifies these slices into a size of 224 × 224. The experiment uses the CAT12 toolkit of the SPM12 software to preprocess the images. Image preprocessing includes format conversion, skull stripping, grayscale normalization, MRI slicing, and uniform sizing, etc. The detailed preprocessing process is shown in Figure 6;
(2) The Keras experimental platform was built and the STN + ResNet + attention network model was designed;
(3) The K-fold (K = 5) cross validation method was used to randomly divide the dataset, with 80% used as the training set and 20% used as the test set;
(4) The training set was input into the network for training and the training results were obtained;
(5) The optimal model parameters were saved and tested in the model using the test set data.

**Figure 7:**
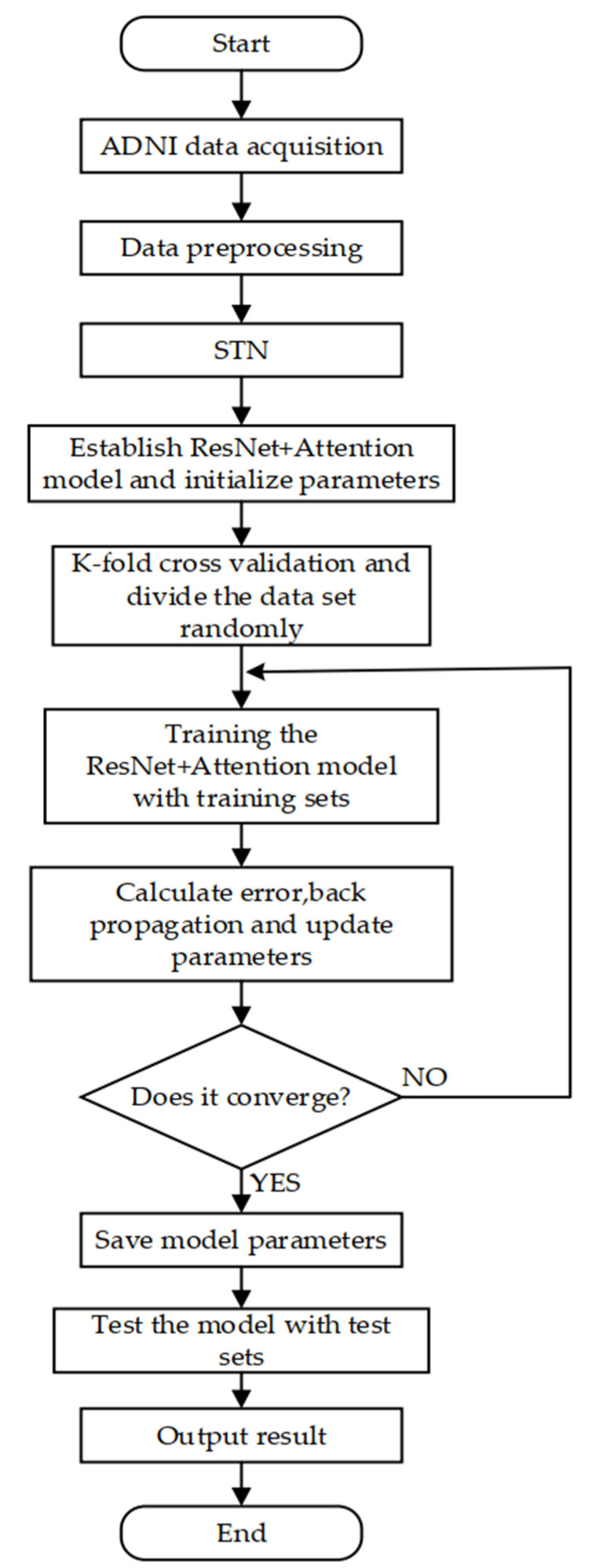
The detailed step flow chart.

The flow chart with detailed steps is shown in Figure 7.

## 4. Experimental Results and Discussion

We considered our result in the context of multi-class classification. The multi-classification data were transformed into two classification problems and a one-vs-rest strategy was adopted—that is, one category comprised positive samples and the other categories comprised negative samples [66,67,68,69,70].

*TP_i_*: the prediction is category *i*, the reality is category *i*.
*TN_i_*: the prediction is other classes of category *i*, the reality is other classes of category *i*.
*FP_i_*: the prediction is category *i*, the reality is other classes of category *i*.
*FN_i_*: the prediction is other classes of category *i*, the reality is category *i*.

Each category was taken as a positive sample to calculate the total accuracy, precision, and recall values for each category. The accuracy can be expressed using Equation (5), as follows:

(5) $$Accuracy=\frac{\mathrm{Number}\;\mathrm{of}\;\mathrm{samples}\;\mathrm{correctly}\;\mathrm{classified}}{\mathrm{Number}\;\mathrm{of}\;\mathrm{samples}\;\mathrm{for}\;\mathrm{all}\;\mathrm{categories}}$$

The precision of a certain category can be understood as predicting the accuracy of the sample, expressed as Equation (6), as follows:

(6) $$Precision_{i}=\frac{TP_{i}}{TP_{i}+FP_{i}}$$

The recall of a certain category can be understood as the extent to which the sample of category *i*, which was correctly predicted, covers the sample of category *i* in the sample set, expressed as Equation (7), as follows:

(7) $$Recall_{i}=\frac{TP_{i}}{TP_{i}+FN_{i}}$$

To investigate the merits and demerits of classifiers under different categories, a macro average should be introduced. Macro-averaging refers to the mathematical average of the values of each statistical index of all types. Their calculation equations are expressed in Equations (8)–(10), as follows:

(8) $$Precision_{macro}=\frac{\sum_{i=1}^{N}Precision_{i}}{N}$$

(9) $$Recall_{macro}=\frac{\sum_{i=1}^{N}Recall_{i}}{N}$$

(10) $$F1_{macro}=\frac{2Precision_{macro}Recall_{macro}}{Precision_{macro}+Recall_{macro}}$$

The total confusion matrix is obtained by adding the values of each folded confusion matrix. For the convenience of calculating the *Precision_macro_*, *Recall_macro_*, and *F1_macro_*, we use the average confusion matrix of multiple classifications. We divide the value of the total confusion matrix by five to get the average confusion matrix. The model proposed in this study was used for training and testing on the selected ADNI dataset, and the test results are shown in Table 3 [70].

For the purpose of illustrating the effectiveness of the method proposed in this paper, ResNet50 baseline, ResNet50 + Mish, and STN + ResNet50 + Mish were selected to conduct experiments using the same dataset. The *Accuracy, Precision_macro_*, *Recall_macro_*, and *F1_macro_* of each model were, respectively, calculated as shown in Table 4.

**Figure 8:**
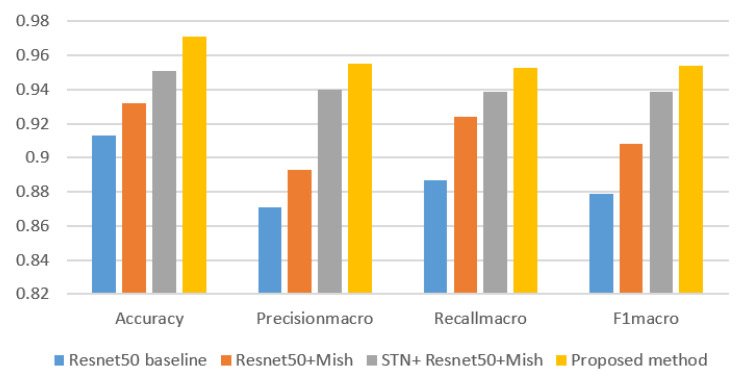
Comparison of the Accuracy, Precision_macro_, Recall_macro_, and F1_macro_ value.

In this paper, the *Accuracy, Precision_macro_*, *Recall_macro_*, and *F1_macro_* value of the above models were successively compared and analyzed, as shown in Figure 8.

The experimental results show that the method proposed in this article is compared with the other three methods in classification accuracy. There is a big improvement, and the standard deviation of the experimental results is smaller. The experiments show that the Mish activation function is used to replace the Relu function in the model, and the accuracy is increased by 1.9% compared with the baseline. After the introduction of the STN and attention mechanism, the accuracy of the model increased by 5.8%.

## 5. Conclusions

In this research article, we propose a deep learning model based on ResNet-50 for the early diagnosis of Alzheimer’s disease. In the model, a new Mish activation function is selected in the ResNet-50 backbone to replace the Relu function, the STN is introduced between the input layer and the improved ResNet-50 backbone, and a non-local attention mechanism is introduced between the fourth and fifth stages of the improved ResNet-50 backbone. The Mish activation function is boundless (that is, the positive value can reach any height) to avoid saturation due to capping. Theoretically, the slight bias toward the negative values allows for better gradient flows in comparison with the hard zero boundary, as in Relu. Integrating the STN module into the ResNet-50 network allows the network to automatically learn how to transform the feature map, thus helping to reduce the overall cost of network training. The addition of a non-local block attention mechanism module provides a solid improvement. The proposed method was validated using the ADNI experimental dataset and compared with the ResNet-50 baseline, ResNet-50 + Mish, and STN + ResNet-50 + Mish models. The experimental results show that the proposed model is more effective and provides a better robustness for clinical application. The integration with STN enhances the ability of this model to extract network features by improving the spatial invariance of the network. This demonstrates its good recognition effect. The introduction of a non-local block attention mechanism can enhance model robustness. In the end, the experiment results found using the ADNI dataset show that the classification accuracy of the algorithm proposed in this paper reached 97.1%, the macro precision reached 95.5%, the macro recall reached 95.3%, and the macro F1 value reached 95.4%, thus verifying the advantages of the proposed model. The proposed method has high significance in the practical application of AD. The combination of AD susceptibility gene detection and pattern recognition is our future research direction [71].

## Figures and Tables

**Table 1:** Information of subjects from ADNI dataset used in this study.

Dataset	ADNI		
Diagnosis	NC	MCI	AD
Number of samples	255	205	55
Number of female samples	127	103	27
Number of male samples	128	102	28
Age	70.6 ± 5.1	73.8 ± 7.5	78.9 ± 8.6
ADAS-Cog	<12	7–17	12–29
MMSE	24–30	23–30	20–26

**Table 2:** The architecture of the proposed method.

Layer Name	Output Size	Layer
STN	224 × 224	Localization network, grid generator, sampler
Conv1	112 × 112	7 × 7, 64, stride 2
Max pooling	56 × 56	3 × 3, stride 2
Stage 2	56 × 56	$\left[\begin{array}{cc} 1\times 1, & 64\\ 3\times 3, & 64\\ 1\times 1, & 256 \end{array}\right]\times 3$, Mish
Stage 3	28 × 28	$\left[\begin{array}{cc} 1\times 1, & 128\\ 3\times 3, & 128\\ 1\times 1, & 512 \end{array}\right]\times 4$, Mish
Stage 4	14 × 14	$\left[\begin{array}{cc} 1\times 1, & 256\\ 3\times 3, & 256\\ 1\times 1, & 1024 \end{array}\right]\times 6$, Mish
Non-local attention module	14 × 14	Attention × 1
Stage 5	7 × 7	$\left[\begin{array}{cc} 1\times 1, & 512\\ 3\times 3, & 512\\ 1\times 1, & 2048 \end{array}\right]\times 3$, Mish
Average pooling	1 × 1	7 × 7, stride 1
FC, softmax	1000-d	

**Table 3:** Average confusion matrix and experimental results.

Confusion Matrix		Predicted Class			Recall	
		NC	MCI	AD		
Actual Class	NC	51	0	0	1.000	*Recall_macro_* = 0.953
	MCI	1	39	1	0.951	
	AD	0	1	10	0.909	
Precision		0.981	0.975	0.909	Acc = 0.971	*F1_macr__o_* = 0.954
		*Precision_macro_* = 0.955				

**Table 4:** Performance comparison of the proposed classification method.

Model	*Accuracy*	*Precision_macro_*	*Recall_macro_*	*F1_macro_*
ResNet50 baseline	0.913 ± 0.035	0.871 ± 0.033	0.887 ± 0.032	0.879
ResNet50 + Mish	0.932 ± 0.032	0.893 ± 0.030	0.924 ± 0.028	0.908
STN + ResNet50 + Mish	0.951 ± 0.021	0.940 ± 0.023	0.939 ± 0.021	0.939
Proposed method	0.971 ± 0.016	0.955 ± 0.015	0.953 ± 0.018	0.954
